# Screening Tools: Their Intended Audiences and Purposes. Comment on “Diagnostic Accuracy of Web-Based COVID-19 Symptom Checkers: Comparison Study”

**DOI:** 10.2196/26148

**Published:** 2021-05-21

**Authors:** Elizabeth Millen, Andreas Gilsdorf, Matthew Fenech, Stephen Gilbert

**Affiliations:** 1 Ada Health Berlin Germany

**Keywords:** COVID-19, symptom checkers, benchmark, digital health, symptom, chatbot, accuracy

We congratulate Munsch et al [1] on their recent publication, “Diagnostic Accuracy of Web-Based COVID-19 Symptom Checkers: Comparison Study.” The study investigated the relative performance of web-based COVID-19 screening tools between April 3 and 9, 2020. It is important to have literature comparing the relative performance of tools; however, it is also important that the approaches used in reports of this type compare “like with like” and are reported rigorously.

There are a number of important limitations in Munsch et al’s [1] paper, the first being that the principal results reported are not fully addressed in the publication, and the second being it is an inappropriate evaluation of COVID-19 screeners designed for use by laypersons at home. COVID-19 screeners were brought into the market at a time of need to lower the burden on health authorities and health care institutions [2]. Overall, they were meant for use at home by people who were either worried about their symptoms or who were anxious about the pandemic in general and in need of reassurance. These people could either be put at ease that their symptoms were likely not caused by COVID-19, or be provided with appropriate localized advice through screeners tailored to their region.

The paper [1] does not investigate the COVID-19 screeners according to their intended purpose and compares tools with fundamentally different intended purposes. The Symptoma tool, developed by the paper’s authors, was designed to use the professional interpretation of both patients’ symptoms and the results of diagnostic tests, such as interpreted CT (computed tomography) images (eg, ground-glass opacities); both provide a predictive likelihood of COVID-19 status. All the other screeners examined in the study were designed for use at home by layperson users, providing a first line of advice to those experiencing possible COVID-19 symptoms. Importantly, none of these tools provide a diagnosis, neither their design concept nor regulatory approval includes this, and testing their diagnostic accuracy is therefore testing something they are specifically labeled as not providing. A meaningful evaluation would be to evaluate the appropriateness of the advice given according to the use context of these screeners (ie, do the screeners provide appropriate information and advice to users based on their symptoms, risk factors, exposure, and their location [including local COVID-19 status and national guidelines]?).

The ability of Symptoma to make use of the professional interpretation of clinical findings might be useful in some hospital settings as a clinical decision support tool, but COVID-19 tests are available in this setting, and at this stage in the patient journey, the tool cannot lessen the burden on the health care system. Based on the arguments above, it is critical to interpret the results in Multimedia Appendices 8-12 [1], which show that Symptoma is not superior in specificity and sensitivity as claimed in the main manuscript, and to remember that the appropriateness of specific advice in a given situation is more important to the user than either specificity or sensitivity.

## Abbreviations

CT: computed tomography

## References

[1] Munsch Nicolas, Martin Alistair, Gruarin Stefanie, Nateqi Jama, Abdarahmane Isselmou, Weingartner-Ortner Rafael, Knapp Bernhard (2020). Diagnostic Accuracy of Web-Based COVID-19 Symptom Checkers: Comparison Study. J Med Internet Res.

[2] Ross C (2021). Symptom checker steers Covid-19 patients to care. STAT.

