# Correction: Screening Tools: Their Intended Audiences and Purposes. Comment on “Diagnostic Accuracy of Web-Based COVID-19 Symptom Checkers: Comparison Study”

**DOI:** 10.2196/31268

**Published:** 2021-06-18

**Authors:** Elizabeth Millen, Andreas Gilsdorf, Matthew Fenech, Stephen Gilbert

**Affiliations:** 1 Ada Health Berlin Germany

In “Screening Tools: Their Intended Audiences and Purposes. Comment on ‘Diagnostic Accuracy of Web-Based COVID-19 Symptom Checkers: Comparison Study’” (J Med Internet Res 2021;23(5):e26148), one error was noted.

In the originally published manuscript, an incorrect statement was included in the Conflicts of Interest section:

EM, MF, and SG are employees of Ada Health GmbH. AG has no conflicts to declare.

The statement has been corrected as follows:

All authors are employees of Ada Health GmbH.

The correction will appear in the online version of the paper on the JMIR Publications website on June 24, 2021, together with the publication of this correction notice. Because this was made after submission to PubMed, PubMed Central, and other full-text repositories, the corrected article has also been resubmitted to those repositories.

